# Inhibition of autophagy by chloroquine prevents resistance to PI3K/AKT inhibitors and potentiates their antitumor effect in combination with paclitaxel in triple negative breast cancer models

**DOI:** 10.1186/s12967-022-03462-z

**Published:** 2022-06-27

**Authors:** Stefania Cocco, Alessandra Leone, Maria Serena Roca, Rita Lombardi, Michela Piezzo, Roberta Caputo, Chiara Ciardiello, Susan Costantini, Francesca Bruzzese, Maria José Sisalli, Alfredo Budillon, Michelino De Laurentiis

**Affiliations:** 1 Department of Breast and Thoracic Oncology, Division of Breast Medical Oncology, Istituto Nazionale Tumori IRCCS “Fondazione G. Pascale”, 80131 Naples, Italy; 2 Experimental Pharmacology Unit, Laboratories of Naples and Mercogliano (AV), Istituto Nazionale Tumori IRCCS “Fondazione G. Pascale”, 80131 Naples, Italy; 3 Animal Facility, Istituto Nazionale Tumori IRCCS “Fondazione G. Pascale”, 80131 Naples, Italy; 4 Department of Neuroscience, Reproductive and Odontostomatological Sciences, University of Naples Federico II, Naples, Italy

**Keywords:** Breast Cancer, TNBC, Autophagy, PI3K/AKT/mTOR inhibitors, Chloroquine

## Abstract

**Background:**

Triple negative breast cancer (TNBC) is an aggressive disease characterized by high risk of relapse and development of resistance to different chemotherapy agents. Several targeted therapies have been investigated in TNBC with modest results in clinical trials. Among these, PI3K/AKT inhibitors have been evaluated in addition to standard therapies, yielding conflicting results and making attempts on elucidating inherent mechanisms of resistance of great interest. Increasing evidences suggest that PI3K/AKT inhibitors can induce autophagy in different cancers. Autophagy represents a supposed mechanism of drug-resistance in aggressive tumors, like TNBC. We, therefore, investigated if two PI3K/AKT inhibitors, ipatasertib and taselisib, could induce autophagy in breast cancer models, and whether chloroquine (CQ), a well known autophagy inhibitor, could potentiate ipatasertib and taselisib anti-cancer effect in combination with conventional chemotherapy.

**Methods:**

The induction of autophagy after ipatasertib and taselisib treatment was evaluated in MDAMB231, MDAM468, MCF7, SKBR3 and MDAB361 breast cancer cell lines by assaying LC3-I conversion to LC3-II through immunoblotting and immunofluorescence. Other autophagy-markers as p62/SQSTM1 and ATG5 were evaluated by immunoblotting. Synergistic antiproliferative effect of double and triple combinations of ipatasertib/taselisib plus CQ and/or paclitaxel were evaluated by SRB assay and clonogenic assay. Anti-apoptotic effect of double combination of ipatasertib/taselisib plus CQ was evaluated by increased cleaved-PARP by immunoblot and by Annexin V- flow cytometric analysis. In vivo experiments were performed on xenograft model of MDAMB231 in NOD/SCID mice.

**Results:**

Our results suggested that ipatasertib and taselisib induce increased autophagy signaling in different breast cancer models. This effect was particularly evident in PI3K/AKT resistant TNBC cells, where the inhibition of autophagy by CQ potentiates the therapeutic effect of PI3K/AKT inhibitors in vitro and in vivo TNBC models, synergizing with taxane-based chemotherapy.

**Conclusion:**

These data suggest that inhibition of authophagy with CQ could overcome mechanism of drug resistance to PI3K/AKT inhibitors plus paclitaxel in TNBC making the evaluation of such combinations in clinical trials warranted.

**Supplementary Information:**

The online version contains supplementary material available at 10.1186/s12967-022-03462-z.

## Background

Breast cancer is a heterogeneous disease, characterized by different clinical outcomes and response to therapy depending on the molecular subtypes [1]. Triple negative breast cancer (TNBC) accounting for about 10% to 15% of all breast cancers, is characterized by an aggressive phenotype, high genomic instability, tendency to develop metastases [2, 3] and chemoresistance [4–7]. Taxanes-based chemotherapy is still the standard of care for the majority of early and advanced TNBCs but clinical outcome is still poor compared to other subtypes, with high risk of relapse relapse and poor survival in the metastatic setting [8]. There is, therefore, an urgent need to identify new molecular targeted treatments for TNBC.

The PI3K/AKT/mTOR pathway plays a major role in human cancers, being involved in the regulation of critical processes such as cell cycle, proliferation, metastatic progression and resistance to antitumor treatments [9, 10]. This pathway comprises a family of intracellular signal transducer enzymes with three key regulatory nodes: PI3K, AKT and mammalian target of rapamycin (mTOR) [1, 11]. PI3K/AKT/mTOR hyperactivation is common finding in cancer, and somatic mutations in PIK3CA/AKT/mTOR axis, like gain-of-function mutations of *PIK3CA* gene and loss/low expression of the regulatory molecule PTEN, have been identified as responsible of resistance to conventional therapeutical regimens in different tumours, including breast cancer [12]. Thus, several agents acting on the PI3K/AKT/mTOR pathway, have been developed and tested in clinical trials in combination with standard therapies [8, 13]. Taselisib, a selective inhibitor of mutant PI3K, and ipatasertib, a selective ATP-competitive pan-Akt inhibitor, have been studied in clinical trials in combination with endocrine agents (NCT02340221, NCT02273973, NCT01296555) or taxanes (NCT02301988, NCT01862081, NCT02162719, NCT03337724), showing conflicting results in patients harboring PI3K/AKT/PTEN-altered tumours [14–18]. Several evidences have showed that common PI3K-AKT/mTOR inhibitors could induce autophagy in different preclinical models, promoting the escape from their antitumor effect. Mechanistically this effect is mediated by the inhibition of mTOR complex 1 (mTORC1)- including mTOR, RAPTOR, PRAS40, DEPTOR, mLST8, Tti1 and Tel2 proteins- that represent the main negative regulator of autophagy induction [19–24].

Autophagy is a complex catabolic process in which cells destroy defective cellular components and recycle their constituting elements to sustain cellular metabolism [25]. The role of autophagy in cancers is controversial as it seems to promote both anti-tumour or pro-tumour pathways, depending on tumor types and stages [26–30]. For example during early carcinogenesis, autophagy might exert an antitumor effect by preventing the genomic instability due to accumulation of damaged proteins and organelles [31–33]. However, during tumour progression, autophagy is able to increase stress tolerance, drug-resistance and tumor cell survival in hostile conditions [34].

Indeed, aggressive tumours, like TNBC, show higher level of autophagy to better tolerate cellular stress occurring during the metastatic process [8, 35]. Therefore, autophagy inhibitors like chloroquine (CQ) and hydroxychloroquine (HCQ) have been largely tested as antitumor agents in preclinical studies, and are currently in development in clinical trials for different cancer types, alone or in combination with standard therapies [36, 37].

The aim of this study was to evaluate the antitumor activity of PI3K or AKT inhibitors plus CQ, in order to prevent authophagy -mediated mechanism of resistance, in combination with taxanes. In details, we showed that, both ipatasertib and taselisib, can induce autophagy in several breast cancer cell lines, characterized by different genetic background, including variable expression of ER/HER2 receptors or mutations of PI3K/AKT pathway. This effect was particularly evident in TNBC where we observed strong potentiation of antitumor activity by combining PI3K/AKT pathway inhibitors plus CQ, and more importantly, where a clear synergistic antitumor effect was observed both in vitro and in vivo in triple combination with paclitaxel.

## Methods

### Cell cultures

Human breast cancer cells MDAMB231, MDAMB468, MCF-7, SKBR3 and MDAMB361 were purchased from American Type Culture Collection (Rockville, MD, USA). All cell lines were genotyped to confirm their origin. MDAMB231, MCF7 and SKBR3 cells were maintained in DMEM high glucose (Lonza) complemented with 10% fetal bovine serum (FBS; Lonza). MDAMB468 and MDAMB361 were maintained in DMEM-F12 complemented with 10% FBS. All media were supplemented with 10000 U/ml penicillin and 10 mg/ml streptomycin (Lonza) and 4 mM L-glutamine in a humidified atmosphere composed of 95% air and 5% CO2 at 37  °C. Cell lines were regularly inspected for mycoplasma.

### Drugs and Reagents

Paclitaxel was purchased from Selleck Chemicals (Selleckchem, Houston, TX, USA) and Chloroquine diphosphate salt from Sigma Aldrich. Taselisib (GDC0032) and ipatasertib (GDC-0068) were supplied by Genentech (Research proposal nr. OR-215703). They were dissolved in sterile dimethylsulfoxide (DMSO) and a 500 mM and 100 mM stock solutions, respectively, were prepared and stored in aliquots at −20 °C. Working concentrations were diluted in appropriate medium.

### Cell proliferation assay and drugs combination studies

Cell proliferation was measured in 96-well plates in cells untreated and treated with ipatasertib, taselisib, paclitaxel and CQ as single agent or in combination. Cell proliferation was measured using a spectrophotometric dye incorporation assay Sulforhodamine B [38] after 48 or 72 h of treatment. IC50 were determined by interpolation from dose-response curves.

### Clonogenic assay

Single cell suspensions were plated at 50–100 cells/well in 12 wells plate. After 24 h, the cells were treated with single or combinations of drugs, daily for 10 days. Colonies were visualized by incubation with 0.5% crystal violet dissolved in 20% methanol for 30 min, and photographed. Then, colonies were dissolved in 100% methanol and quantified by spectrophotometry.

### Spheroid-forming assay

Spheroids were cultured as described before [39] in appropriate Sphere Medium. The cells (40,000 cells/ml) were plated in low attachment multiwell plates and treated with indicated drugs. Spheroids have been treated as reported in figures. Spheroids were scored with CellTiter- Glo^®^ 3D Cell Viability Assay (Promega, Madison, WI, USA).

### Protein extraction and Western blotting

Cells, grown and treated as indicated in results, were washed once with ice-cold PBS and centrifuged. The cell pellet was lysed by Nonidet P40 plus protease inhibitors (Thermo Fisher Scientific, Waltham, MA USA) and clarified by centrifugation. Equal amount of protein, monitored by Bradford assay, was separated on 8–10% Sodium Dodecyl Phosphate (SDS) polyacrylamide gel electrophoresis (PAGE) Enhanced chemiluminescence (ECL) immunodetection reagents were from GE Healthcare. Image Quant LAS 500, and ImageQuantTL image software (GE Healthcare) were used to detect chemiluminescence and quantify signal, respectevely. Image J was used to quantify protein bands from western blot images. The quantification reflects the relative amounts as a ratio of each protein band relative to loading control (β-actin). The following antibodies were used: polyclonal LC3B Antibody #2775 (1:1000) Cell Signaling, polyclonal anti PARP#9532S (1:1000) Cell Signaling, polyclonal anti p62/SQSTM1 #J PM045 MBL international (1:1000), polyclonal anti ATG5 PM050 MBL International (1:1000), monoclonal Anti-β-Actin A5316 (1:1000) Sigma Aldrich.

### Immunofluorescence assay

Cells, plated on slides in 24-wells plate at 25000-50000 cells/well, were treated with drugs as indicated in figure legends. Then cells were fixed in 4% paraformaldehyde (20 min at RT), blocked by 0.2% PBS/BSA solution (1h at RT) and incubated with primary (1:100) anti-LC3 or anti-p62/SQSTM1 antibodies overnigh at 4°. After washes, cells were incubated with (1:200) anti-rabbit Alexa Fluor-488 or Alexa Fluor-594 for 1h at RT, mounted on slide holder using mountant medium (Life technologies, Gaitherburg, MD, USA). Confocal images were obtained using Zeiss inverted 700 confocal laser scanning microscopy and a ×63 oil immersion objective.

### Flow cytometry analysis of apoptosis

Cells were treated with the drug combinations as indicated in relative legends. Apoptosis was measured after staining with annexin V-fluorescein isothiocyanate (annexin V-FITC). Annexin positive cells were quantified with FACS calibur flow cytometer (Becton Dickinson), analysed using CellQuestPro software (Becton Dickinson). Data were acquired after analysis of at least 10,000 events [40].

### Flow cytometry analysis of cell cycle

Analysis of cell cycle kinetic was performed after treatment as indicated in the relative legend. Briefly, cells were fixed in 70% ethanol, stained with propidium iodide and evaluated by a FACScalibur flow cytometer (Becton Dickinson). For each sample, 20,000 events were collected. Cell cycle analysis was performed with ModFit LT software (Verity Software House, Inc., Topsham, ME). FL2 area versus FL2 width gating was done to exclude doublets from the G2-M region.

### In vivo experiments

Female NOD/SCID athymic mice (Charles River, Wilmington, MA, USA) were acclimatized in the Animal Care Facility of “Fondazione G.Pascale-IRCCS-CROM Laboratories, in accordance with ”Directive 2010/63/EU on the protection of animals used for scientific purpose” and made effective in Italy by the legislative DLGS 26/2014. The study was approved by the Italian Ministry of Health. MDAMB231 cells (7 × 10^6^) diluted in 200 μl [PBS/Matrigel GF (Becton Dickinson) 1/1] were injected subcutaneously (s.c) in the flank regions of the mice. After tumors reached approximately 100 mm^3^, mice were randomized into treatment arms with 5–7 tumors per group. Taselisib (5 mg/kg) was dissolved in a vehicle containing 0.5% methylcellulose with 0.2% TWEEN-80 and was administered via daily oral gavage [41]. Paclitaxel were diluted in physiological solution and administrated intraperitoneally (i.p.) weekly. CQ were dissolved in physiological solution and administered daily via oral gavage. In particular, taselisib 5 mg/kg was administrated x os/daily (5 days/week) by gavage, CQ (30 mg/kg) was administrated x os/daily (5 days/week) by gavage and paclitaxel 10mg/Kg was administrated once a week intraperitoneally (IP). Treatment lasted 2 weeks followed by 1 week of follow-up.

Mice in the control groups were treated with relative vehicles via daily oral gavage and weekly IP. Tumor volume (TV) (mm3), Tumor growth delay (TGD) and the percent change in the experimental groups was compared with that of the vehicle control groups as described before [42]. Tumor incidence curves to analyze tumor engraftment (first appearance of a palpable mass) was performed taking advantage of Kaplan-Meier approach. Tumor size was measured twice a week and calculated as: ½ × width2 × length. Animals were monitored for abnormal tissue growth and euthanized if excessive health deterioration was observed.

### Plasmid transfection

MDAMB231 cells were plated on slides in 24-wells plate at 25000 cells/well. Then they were transfected with EGFP-LC3 plasmid using Lipofectamine 2000 Reagents (Invitrogen, Carlsbad, CA, USA), according to the manufacturers recommendation [43]. 12h after the transfection, cells were treated with ipatasertib, taselisib or CQ at concentrations and time indicating in the relative legend and processed for immunofluorescent experiments as described before. EGFP-LC3 plasmid was purchased by Addgene plasmid #11546 (http://n2t.net/addgene:11546; RRID: Addgene_11546).

### Statistical analysis

All experiments were performed at least three times. Statistical significance was determined by the one-way ANOVA, Tukey’s multiple comparison test, Dunn’s multiple comparisons test and Log Rank test; a p<0.05 was considered to be statistically significant and the specific values are reported or indicated in legends to figures as *, p< 0.05; **, p< 0.01; ***, p< 0.001. All statistical evaluations were performed with GraphPad Prism 8.

## Results

### Ipatasertib and taselisib induced antitumor effects in breast cancer cell lines depending of their molecular profile

We screened the antitumoral activity of ipatasertib and taselisib in five breast cancer cell lines, MDAMB231, MDAMB468, MCF7, SKBR3 and MDAMB361, each one having different expression of ER and HER2 receptors, or mutations involving PI3K/AKT and BRAF/RAS pathways. As reported in Table 1, the sensitivity to both drugs was dependent on the molecular subtypes. In detail, cells with HER2 amplification with or without PI3KCA mutation, such MDAMB361 and SKBR3, were the most sensitive to both drugs, in line with previous observations [44]; cell lines with gain of function mutations of PI3KCA, such as MCF7 and MDAMB361, responded preferably to taselisib, as widely demonstrated before [45]. TNBC cells, MDAMB231 and MDAMB468, resulted the most resistant, however, the EGFR amplification along with PTEN-null mutation in MDAMB468 cell line [46] makes them more sensitive to PI3K/AKT inhibition compared with K-RAS-mutated MDAMB231 cell line [13, 47, 48]. Overall, taselisib was more effective than ipatasertib to reduce cell proliferation in all evaluated breast cancer cell lines.

**Table 1:** Genetic background of breast cancer cell lines and antiproliferative effect of drugs alone

CELL LINE	ER amplification	HER2 amplification	Mutations	Additional features	IPATASERTIB IC50	TASELISIB IC50
MDAMB231	−	−	KRAS/B-RAF	p53 NF2 p16 CDKN2 p14 CDKN2A	70 µM	25 µM
MDAMB468	−	−	PTEN null	EGFR ampification p53 SMAD4 RB1	5 µM	2.5 µM
MCF7	+	−	PI3KCA	p16 CDKN2 p14 CDKN2A	10 µM	500 nM
SKBR3	−	+	wt	p53	500 nM	50 nM
MDAMB361	+	+	PI3KCA	NA	1 µM	100 nM

**Fig. 1:**
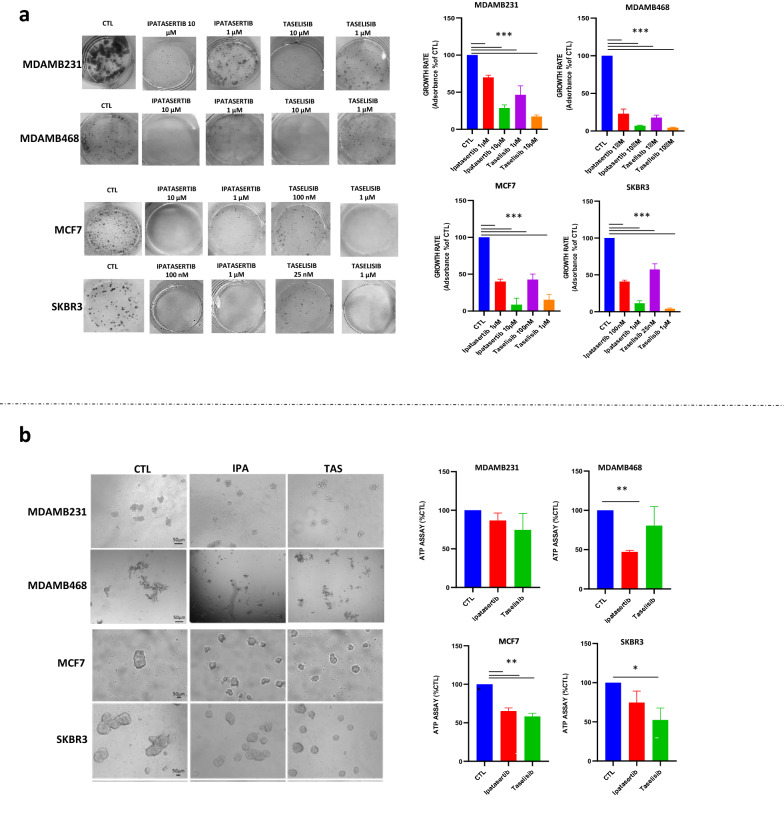
Ipatasertib and taselisib reduce cell proliferation in breast cancer models. **a** and ability to prevent clonogenic formation after daily administration for 10 days of IC30 doses for each drug or at fixed drug dose of 1 µM, expressed as % of CTL. Each experiment is representative of three independent experiments. **b** Ipatasertib and taselisib treatment impair 3D tumor spheroid derived from breast cancer cells. Representative images of first generation 3D tumor spheroid, exposed to ipatasertib and taselisib, administrated to IC30 doses for 72 h. Tumor cell growth was reduced in MCF7 and SKBR3 while MDAMB231 and MDAMB468 cells form only aggregates. Quantification of ATP was used to measure reduction of cellular growth, expressed as % of CTL. Each experiment is representative of three independent experiments. Statistically significant results are reported (*** indicates P < 0.0005, ** indicates P < 0.005 and * indicates P < 0.05)

These data were confirmed by clonogenic assay where we treated the cells with either different doses depending on the data from antiproliferative assays described above or with a fixed dose of 1µM for each drug, in order to compare the sensitivity among the different cell lines. As shown in Fig. 1a and Additional file 1: Fig. S1a, long term exposure up to 10 days was effective to prevent clonogenic formation in all cell lines, including resistant TNBC cell line MDAMB231.

Furthermore, we assessed the antitumor effect of ipatasertib and taselisib by evaluating cell-cycle perturbation and apoptosis induction, confirming that both drugs could cause G1 phase accumulation (Additional File 1: Fig. S2a) and/or pro-apoptotic effect (Additional file 1: Fig. S2b) depending on cell line, as previously described [47].

Finally, to better recapitulate tumor growth complexity [49], we next tested the efficacy of ipatasertib and taselisib to form breast cancer cells 3D self-assembled spheroids in low attach condition. We observed that MCF7, SKBR3 and MDAMB361 were able to form spheroids while the two TNBC cell lines, MDAMB231 and MDAMB468, produced only loose aggregates. Furthermore, by using an ATP-based vitality assay we observed that both drugs prevented the spheroid formation in all cell lines, confirming a preferential inhibitory activity of taselisib in MCF7, SKBR3 and MDAMB436 cell lines and of ipatasertib in MCF7 and MDAMB468 cell lines (Fig. 1b and Additional file 1: Fig. S1b).

Antitumoral activity of ipatasertib and taselisib has been tested in five different breast cancer cell lines, MDAMB231, MDAMB468, MCF7, SKBR3 and MDAMB361 showing different molecular profile as reported. Ipatasertib and taselisib administered as single agent, was able to inhibit tumor growth (IC50 of each drug was determined by SRB assay at 48h in MCF7, SKBR3, and 72h in MDAMB361, MDAMB231 and MDAMB468).

### The inhibition of PI3K/AKT/mTOR pathway by ipatasertib and taselisib was paralleled by autophagy induction

**Fig. 2:**
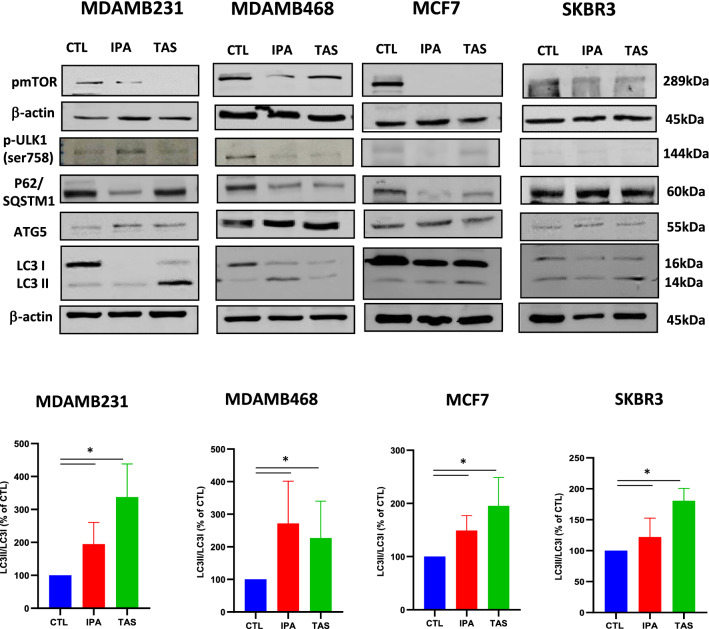
Ipatasertib and taselisib treatment induces Autophagy in all breast cancer analyzed. **a** The exposure to fixed dose (IC50) of ipatasertib and taselisib induces the reduction of expression of phospho-mTOR, associated with the increase of autophagy signaling, as showed by increase of LC3 II/LC3 I ratio by immunoblot assay by reduction of p62 and p-ULK1-ser758, or increase of ATG5 after 24 h of exposure in MCF7, SKBR3, MDAMB231 and MDAMB468 cell lines. In MDAMB231 cell line the maximum dosage of 10 µM was used for both drugs. Statistically significant results are reported (*** indicates P < 0.0005, ** indicates P < 0.005 and * indicates P < 0.05)

Both ipatasertib and taselisib are able to trigger a strong reduction of activity of PI3K/AKT/mTOR pathway with decreased mTOR phosphorylation [50]. On the other hand several preclinical evidences have shown that drugs targeting PI3K/AKT/mTOR pathway can induce autophagy by inhibition of mTORC1 [11]. Therefore, we next investigated if ipatasertib and taselisib could induce autophagy in our breast cancer cell models. Indeed, in all breast cancer cell lines, both drugs determined the inhibition of phospho-mTOR protein expression, paralleled to a significant induction of autophagy, as shown by western blot analysis and quantification of the LC3-I conversion to the lipidated, autophagosome-associated form, LC3-II (Fig. 2 and Additional file 1: Fig. S1c).

We confirmed authophagy induction by also evaluating the expression of other autophagic biomarkers such as p-ULK1-ser758, a phosphorylation induced by p-mTOR, ATG5 a protein critical for autophagosome formation [51] and SQSTM1/p62 (p62 protein), a specific cargo protein of autophagosomes which forms aggregates prior to transport into the lysosomes [52]. In detail, both ipatasertib and taselisib induced decrease of SQSTM1/p62 and p-ULK1-ser758, or increase of ATG5, particularly in the PI3K/AKT-inhibitors resistant TNBC cells, MDAMB231 and MDAMB468 (Fig. 2a and Additional file 1: Fig. S1c). This effect was more pronounced in the taselisib-treated MDAMB231 cells and in both taselisib- and ipatasertib-treated MDAMB468 cells.

**Fig. 3:**
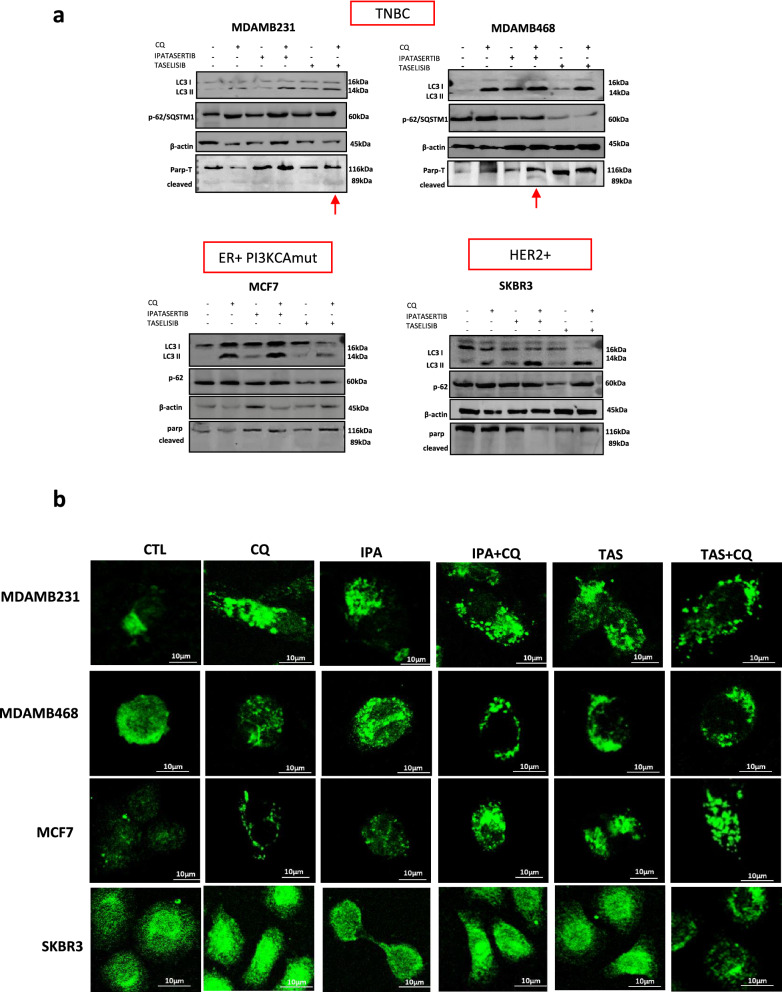
Chloroquine reduces autophagic flux and induce apoptosis in TNBC cell lines. **a** The addition of CQ 10 µM to ipatasertib and taselisib (IC50) induces accumulation of LC3II and p62 protein expression after 24 h, due the reduction of autophagic flux, while expression of cleaved parp was increased in taselisib + CQ group in MDAMB231 cell line and in ipatasertib + CQ group in MDAMB468 cell line. **b** Representative confocal images of cell lines immuno-stained with anti-LC3IIb antibody reveals accumulation of autophagosomes (green dots) in treatments with ipatasertib, taselisib, CQ or combinations, due to the induction of autophagy or reduction of autophagic flux exerted by CQ. In MDAMB231 cell line the maximum dosage of 10 µM was used for both drugs. Statistically significant results are reported (*** indicates P < 0.0005, ** indicates P < 0.005 and * indicates P < 0.05)

In order to functionally confirm that authophagy induction represents a mechanism of resistance against both ipatasertib and taselisib we took advantage of CQ, that block the autophagic flux at late stage by inhibiting the fusion with lysosomes or by blocking lysosomal degradation [53], thus determining the accumulation of autophagic machinery such as LC3-II and SQSTM1/p62 proteins in treated cells. As shown in Fig. 3a and Additional file 1: Fig. S1d, the combination of CQ plus either ipatasertib or taselisib induced a more robust accumulation of LC3 and p62/SQSTM1 co-aggregates in all breast cancer models. More importantly, combination treatment induced proapoptotic effect, as demonstrated by increased cleaved-PARP expression in resistant MDAMB231 cells (taselisib+CQ) and in MDAMB468 (ipatasertib+CQ).

Moreover, we confirmed the presence of autophagic vacuoles in ipatasertib and taselisib-treated cells, by evaluating endogenous LC3-II by immunofluorescence. As reported in Fig. 3b and Additional file 1: Fig. S1e, 24h of exposure with either ipatasertib or taselisib caused an accumulation of LC3-II positive aggregates that appeared as distinct puncta, less pronounced in highly sensible cell lines such as SKBR3 and MDAMB361. Notably, the co-treatment with CQ caused the accumulation of LC3-II puncta confirming authophagy flux block. These data were further confirmed by analyzing the concomitant expression of LC3-positive structures and SQSTM1/p62 protein in MDAMB231 cells overexpressing the EGFP-tagged LC3 construct (Additional file 1: Fig. S3). In detail, either ipatasertib or taselisib treatment caused a rilevant increase of LC3 puncta compared to the control group, while co-treatment with CQ led to a pronounced accumulation of p62-positive aggregates that appeared to co-localize with LC3. Overall these data demonstrated for the first time that both ipatasertib and taselisib are able to induce autophagy, particularly in TNBC models, and that the concomitant treatment with CQ inhibit this process also potentiating the pro-apoptotic effect induced by these agents.

### Chloroquine, by inhibiting authophagy, potentiates the antitumor effect of ipatasertib and taselisib, in TNBC cells

Since autophagy confer stress tolerance to mantain tumor cell survival upon PI3K-AKT/mTOR inhibitors treatment [4, 23, 49] we next tested if CQ was able to potentiate the antitumor effect of both ipatasertib and taselisib in TNBC MDAMB231 and MDAMB468 cells.

**Fig. 4:**
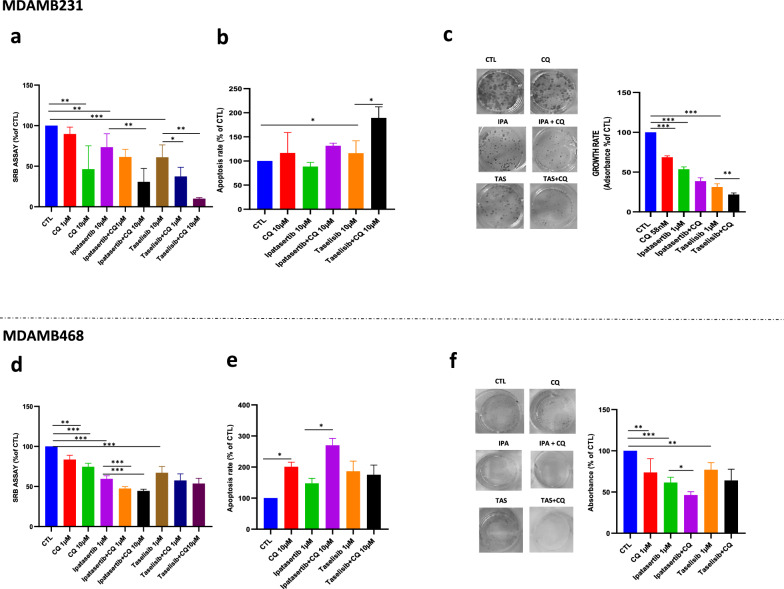
Chloroquine potentiates the antitumor effect of ipatasertib and taselisib in TNBC cells **a-d** The addition of CQ 1-10 µM to ipatasertib and taselisib inhibits cell proliferation in MDAMB231 and MDAMB468 cell lines, measured by SRB assay after 72 h and expressed as % of CTL. Each experiment is representative of three independent experiments. **b** By citofluorimetric assay, CQ 10 µM in combination with taselisib 10 µM, increases apoptosis, as measured by enhancement of Annexin V-FITC and Propidium Iodide, after 24 of treatment in MDAMB231, expressed as % of CTL **e** CQ alone or in combination with ipatasertib 1 µM, increases apoptosis, as measured by enhancement of Annexin V-FITC and Propidium Iodide, after 48 h of treatment in MDAMB468, expressed as % of CTL. Each experiment is representative of three independent experiments. **c** Daily exposure for 10 days of low dose (58 nM) of CQ reduces clonogenic proliferation of MDAMB231 cell line alone or in combination with ipatasertib and taselisib (1 µM). Cell growth was represented as % of CTL. **f** Daily exposure for 10 days of low dose (1 µM) of CQ reduces clonogenic proliferation of MDAMB468 cell line in combination with ipatasertib and taselisib (1 µM). Cell growth was represented as % of CTL. Each experiment is representative of three independent experiments. Statistically significant results are reported (*** indicates P < 0.0005, ** indicates P < 0.005 and * indicates P < 0.05)

The co-administration of CQ with either ipatasertib and taselisib potentiated the antitumor effect of both agents as shown by SRB antiproliferative assay (Figs. 4a, d), apoptosis evaluated by assay Annexin V- flow cytometric analysis (Fig. 4b, e), and clonogenic assay (Fig. 4c, f). In particular, the synergistic cytotoxic effect between CQ and taselisib was more pronounced in MDAMB231, while in MDAMB468 cell line CQ synergized preferably with ipatasertib. Notably, we showed synergistic antitumor effect by using low dosages of CQ consistent with the plasma concentrations reached by the clinical use of the agent in rheumatoid arthritis (3.6 mg/kg) [54] and malaria patients (250 mg up to 500 mg daily) [55], suggesting the potential safety usage of CQ in anticancer combination therapeutic approach. The combined treatment of CQ plus ipatasertib or taselisib was also evaluated in the other breast cancer cell lines, where we observed that higher doses of CQ (10-20 µM) were needed to gain a significant synergistic antitumor effect, as shown by short-term antiproliferative assay (Additional file 1: Fig. S4a) or by cytotoxic assay, measuring LDH release (Additional file 1: Fig. S4b). Overall these data strongly support the pro-survival role of autophagy against the antitumor effect of PI3K/AKT inhibitors, particularly in TNBC cell lines, supporting a combination approach with CQ to bypass autophagy-related drug resistance mechanisms.

### In vitro* and *in vivo* synergistic antitumor effect of chloroquine plus taselisib and paclitaxel triple combination in TNBC cells*

**Fig. 5:**
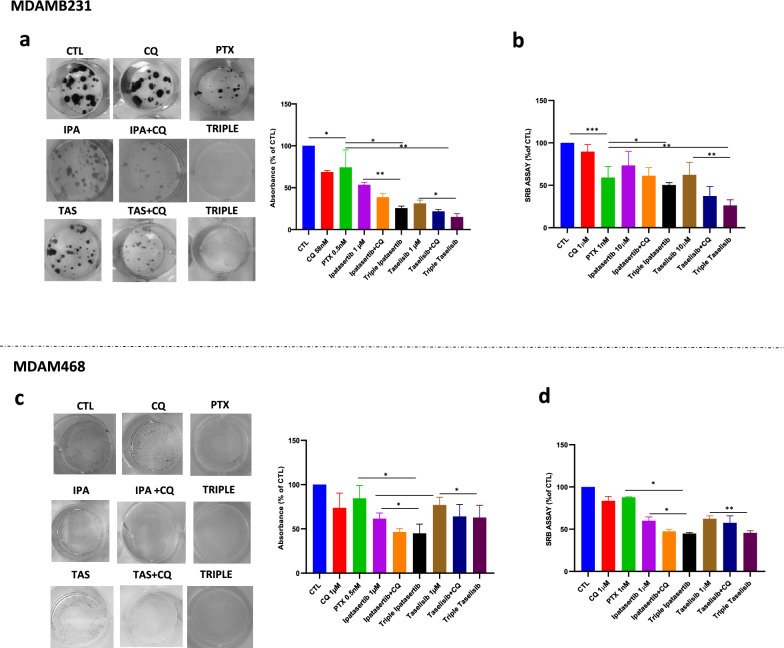
Chloroquine potentiates the antitumoral activity of chemotherapy and PI3K/AKT inhibitors combination in TNBC cell lines **a-c** Daily exposure for 10 days to triple combination of low doses of CQ (58 nM-1 µM), paclitaxel (0,5 nM), ipatasertib (1 µM) or taselisib (1 µM) exposure, prevent clonogenic formation in MDAMB231 and MDAMB468 cell lines. Representative experiments, similar experiments yeld similar results **b-d** Triple combination of low doses of CQ (1 µM), paclitaxel (1 nM), ipatasertib (1–10 µM) or taselisib exposure, to the indicated treatments, induces the inhibition of cell proliferation in MDAMB231 and MDAMB468 cell lines, measured by SRB assay after 72 h and expressed as % of CTL. Each experiment is representative of three independent experiments. Statistically significant results are reported (*** indicates P < 0.0005, ** indicates P < 0.005 and * indicates P < 0.05)

Since chemotherapy is still the standard of care in many early or advanced TNBC tumors, we considered to evaluate the synergistic effect of triple combination of low doses of CQ, plus paclitaxel and either ipatasertib or taselisib. We observed that daily exposure, over 10 days, of low doses of CQ, paclitaxel and either ipatasertib or taselisib caused a severe reduction of both long-term clonogenic and short-term antiproliferative activity activity in MDAMB231 cell line (Fig. 5a–b). Since CQ is not a specific autophagy inhibitor but it can affect other cellular processes beyond autophagy, we silenced ATG5 protein by transfecting MDAMB231 cells with ATG5-siRNA and relative scramble construct (Additional file 1: Fig. S5a) observing similar results on proliferation in ATG5-silenced combinations (Additional file 1: Fig. S5b). Triple combination effects, although less potent, were also observed in MDAMB468 cell line, preferably in combinations with ipatasertib (Fig. 5c–d).

**Fig. 6:**
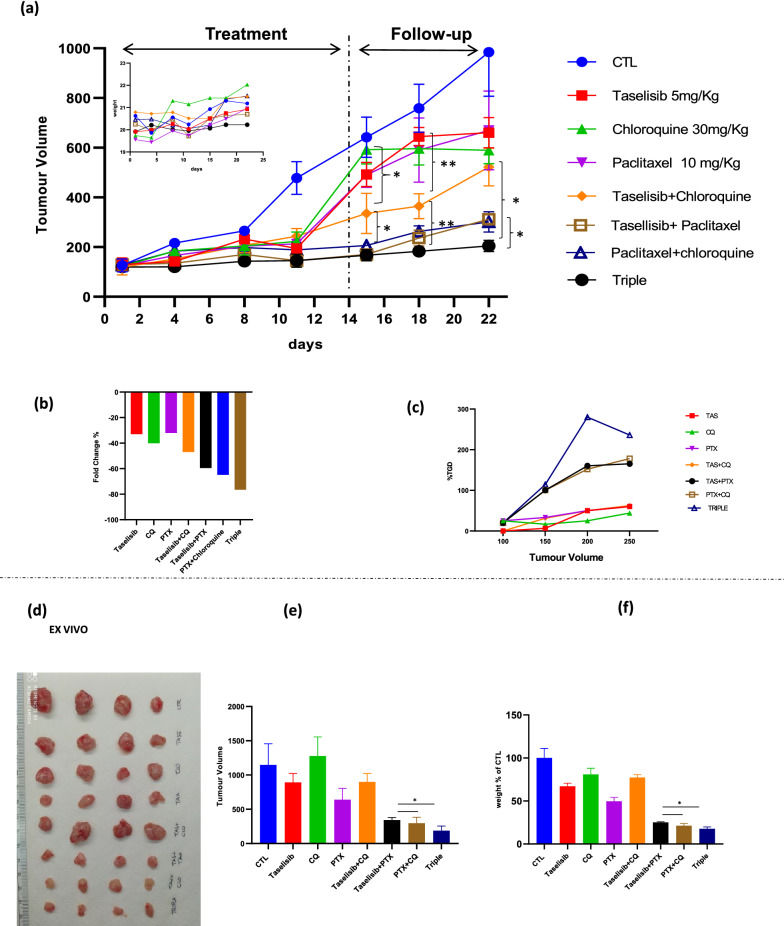
Combination of CQ and taselisib potentiates paclitaxel antitumor effect in *vivo* TNBC xenograft models.** a** MDAMB231 cells (7 × 10^6^) were s.c. injiected in NOD/SCID mice as described in Methods section. When established tumors were palpable, mice were treated with 1) vehicles (CTL group) 2)taselisib (5 mg/kg/os) daily 3)CQ (30 mg/kg/os) daily 4)paclitaxel (10 mg/Kg/IP) weekly 5)taselisib + CQ daily and 6)taselisib + paclitaxel 7)paclitaxel + CQ 8)triple combination. Treatment lasted 2 weeks followed by 1 week of follow-up. Relative tumor volume curves are represented as means ± SE measured at pre-specified time points. Inset, body weight have been measured two times/week. Statistically significant results are reported (*** indicates P < 0.0005, ** indicates P < 0.005 and * indicates P < 0.05). **b** Tumour volume averages from each group at day 0 and day 22 were compared and presented as percentages of vehicle. **c** Tumor growth delay (TGD) determined as %TGD = [(T − C) /C] × 100, where T and C are the mean times expressed in days for the treated or control groups, respectively, to reach a defined tumor volume (see Materials and Methods). **d** Representative images of tumors collected ex vivo on day 28 **e** Tumor volume ex vivo on day 28 represented as means ± SE (f) Tumor weight ex vivo on day 28 represented as means ± SE

Then, to confirm that our novel combination of PI3K/AKT inhibitor plus CQ could potentiate chemotherapy effect, we tested triple combination of taselisib, CQ and paclitaxel in MDAMB231 xenograft model. In details, 45 mice were injected with MDAMB231 cells, and, 6 days after implantation, they were randomly assigned to 8 groups to receive low dosages of CQ, taselisib, paclitaxel, taselisib+CQ, taselisib+ paclitaxel, paclitaxel+CQ, triple combination, or their vehicles. As shown by tumor growth curves (Fig. 6a) triple combination treatment produced a clear statistically significant tumor growth inhibition compared with control, single or double combination treatments groups. Triple combination did not induce any toxicity as reported by maintenance of body weight (inset in Fig. 6A) and the absence of other toxicity signs. In particular, the effect of triple combination was maintained during the follow up period suggesting potential long term effect on tumor regression. Moreover, a reduction of tumor burden by about 80% has been shown only in triple combination group, as highlighted by calculating the percent change in tumor volume from the time of initial treatment (day 1) to the end of the study (day 22) (Fig. 6b). Furthermore, evaluation of the TGD demonstrated that it achieved an apex of more than 250% in the mice treated with triple combination, with mean rate of tumor growth four fold lesser than control group (Fig. 6c). These data were finally confirmed through the measurement of tumor volume (Fig. 6 d–e) and tumor weight (Fig. 6f) ex vivo. Overall we confirmed the capacity of CQ plus PI3K/AKT inhibitors to potentiate chemotherapy efficacy in TNBC models.

## Discussion

TNBC have a very poor clinical outcome and taxanes-based chemotherapy still represent the main standard of care option for this disease. Only recently the checkpoint inhibitor atezolizumab was approved, in combination with nab-paclitaxel in the treatment of PD-L1+ metastatic TNBC patients [56]. PI3K/AKT/mTOR pathway is frequently activated in TNBC, through gain of function mutation of PI3KCA and of loss of function of PTEN [8, 57], and, therefore, the pharmacological inhibition of PI3K/AKT pathway can, in theory, represent a successful treatment strategy in these tumours [58]. In agreement with this hypothesis, in a phase II trial, the TBCRC 032 IB/II trial, taselisib combined with enzalutamide, increased clinical benefit of AR+ TNBC patients [17]. Furthermore, two randomized phase II studies, namely, the Lotus trial [16] and the PAKT trial [59] have showed that adding an AKT inhibitor, ipatasertib or capivasertib, respectively, to paclitaxel, improves progression free survival (PFS) in TNBC patients harbouring activating mutations of PI3K/AKT1/PTEN axis. However, these promising results have not been confirmed in phase III trials [18]. A possible explanation of these conflicting results is that the efficacy of PI3K/AKT inhibitors may be limited by the autoinduction of autophagy, which, in turn, may mediate the development of resistance [25]. Based on this, we speculated that the pharmacological inhibition of autophagy, obtained with CQ administration, could potentiate antitumor effects of ipatasertib/taselisib in combination with conventional chemotherapy. We investigated this hypothesis in breast cancer models characterized by different ER+ HER2+ receptors profile and mutations of PI3KCA/PTEN or KRAS/B-RAF.

Sensitivity to both ipatasertib and taselisib was more evident in HER2+ cell lines and in PI3KCA mutated cell lines, thus confirming previous preclinical studies [48, 56, 60–62], while TNBC cell lines resulted, overall, less sensitive to both drugs, in keeping with previous findings that TNBC tumours are associated with resistance to PI3K/AKT inhibitors [13]. However, as expected, this effect was less evident in PTEN-null and EGFR overexpressing MDAMB468 cell line, compared to KRAS/BRAF-mutated MDAMB231 [46, 47]. These results are consistent with the results of phase Ib clinical trial NCT01791478 showing that a small number of patients (~10%) with KRAS mutations do not derive clinical benefit by alpelisib, an α-selective PI3K inhibitor, in combination with letrozole in ER+/HER2- metastatic breast cancer [62], and of Poseidon phase 1b trial [45], in which mutations of KRAS in circulating tumor (ct) DNA identified patients with clinical resistance to taselisib [63].

In agreement with our hypothesis, we observed that the antitumor effect of both ipatasertib and taselisib was paralleled by the induction of autophagy signaling in all breast cancer cell lines. Recently, Zhai et al. have shown that ipatasertib is able to induce autophagic cell death in hepatocellular carcinoma [64], while, Zorea et al showed that an ovarian cancer cell line undergoes autophagy, after taselisib treatment, to avoid cell death [65]. To our knowledge, our study is the first to show that ipatasertib and taselisib are able to induce autophagy in different breast cancer models. Of note, the increase of autophagy signaling was more evident in the most resistant TNBC KRAS/BRAF-mutated MDAMB231 cell line. This is consistent with other evidences showing that KRAS mutations could drive increased autophagy flux making KRAS mutant models highly sensitive to autophagy inhibition [66–68]. Indeed, in this cell line, the pharmacological inhibition of autophagy, exerted by CQ, significantly increased the efficacy of PI3K/AKT inhibitors, suggesting that the activation of autophagy in this highly aggressive tumours, could represent a mechanism of escape to drug therapy. On the opposite, in the more sensitive breast cancer cell lines, the synergistic antitumor interaction between CQ and the two PI3K/AKT inhibitors was less evident. Overall, in our models, TNBC cell lines appeared strongly autophagy-addicted, indeed the addition of chloroquine to ipatasertib or taselisib caused a strong growth reduction, with a significant increase of apoptosis. The proliferation rate was dramatically reduced with low doses of CQ, while higher doses were requested to achieve a significant reduction of proliferation in the other cell lines.

Other authors have found that TNBC show higher level of autophagy than other breast cancer subtypes. Autophagy proteins, such as Beclin-1 and LC3A/B were found over-expressed in TNBC cells compared to the other breast cancer subtypes [7], and this expression appeared correlated with tumour progression and poor outcome in TNBC [69]. Claude-Taupin et al. found high expression of ATG9 protein in TNBC breast cancer tissues, while the inhibition of ATG9 by shRNA- and CRISPR/Cas9-driven of ATG9A was associated with a regression of pro-cancer phenotypes in a TNBC in vitro model [70]. Hamurcu et al. highlighted that silencing of LC3 and Beclin-1 genes, thereby inhibiting autophagy, significantly suppressed cell proliferation, colony formation, migration and invasion of TNBC models, and induced increase of apoptosis [71]. Accordly, Maycotte et al. showed that silencing of ATG5, ATG7 and Beclin1 reduced the proliferation of different TNBC cell lines (basal and claudin-low) [72]. Finally, Chen et al. showed that the activation of autophagy signaling, was associated with metastatic progression in TNBC models [73]. These data strongly suggest a pro-survival role of autophagy in TNBC tumors.

Based on these findings, in last years, several studies focused on modulation of autophagy in cancer, through the use of well-know molecules such as CQ and HCQ [54]. Preclinical evidences support CQ and HCQ use as anti-cancer therapies, especially in combination with conventional anti-cancer treatments, since they seem to be able to sensitize tumour cells to a variety of drugs, potentiating their therapeutic activity [37]. It has been fully described that CQ and HCQ exert anticancer effects due to their anti-autophagy activities, although other anti-cancer activities, such as modulation of inflammatory pathway and apoptosis, have been highlighted [74, 75].

Several clinical trials are investigating the use of CQ or HCQ, alone or in combination with standard therapies, in different cancer types including breast cancer [25, 37]. Recently, Arnaout et al. published results from a randomized, double-blind clinical trial evaluating treatment with single-agent CQ 500 mg daily for 2–6-weeks prior to breast surgery. The treatment was not associated with any significant effects on breast cancer cellular proliferation, however, it was associated with toxicity that may affect its broader use in oncology [76]. These disappointing results, however, could be in part ascribed to the choose of the wrong target population and the wrong dose [55, 77]. In our study, because of the potential toxicity of CQ, we decided to use low doses of CQ. In particular, in the acute administration the maximum concentration of 10 µM was used while in the chronic administration we used lower concentrations such as 1 µM or 58nM, resembling concentrations that are normally used in clinical practice to treat diseases other than cancer [54, 55]. Interestingly, these concentrations were sufficient to synergize with either taselisib or ipatasertib in TNBC models. Finally, we translated our in vitro observations in a proof-of-concept preclinical *in vivo* study by evaluating CQ plus taselisib in combination with paclitaxel, the standard first line chemotherapy backbone in TNBC. Interestingly, in MDAMB231 cells, the triple combination of low doses of taselisib, CQ and paclitaxel strongly synergized in short and long term exposure, achieving surprising results in in vivo models, where tumor growth was deeply inhibited both during the treatment and the follow-up period, suggesting a carry-over effect lasting even without drug-pressure.

## Conclusions

In summary, we suppose that chronic use of CQ could prevent drug resistance phenomena, mostly in TNBC highly autophagy-addicted tumors. These results strongly support a clinical trial investigating the combination of low doses of CQ plus PI3K inhibitors and chemotherapy, allowing in the next future, an improvement of combinatorial strategies with chemotherapy in TNBC tumours.

## Supplementary Information

**Additional file 1.** **Fig. S1. Ipatasertib and taselisib determines antitumor effects in MDAMB361 cells.** **a** Ipatasertib and taselisib reduce cell proliferation and ability to prevent clonogenic formation after daily administration for 10 days of IC30 doses for each drug or at fixed drug dose of 1µM, expressed as % of CTL. Each experiment is representative of three independent experiments. **b** Ipatasertib and taselisib treatment impair 3D tumor spheroid derived from MDAMB361 breast cancer cells. Representative images of first generation 3D tumor spheroid, exposed to ipatasertib and taselisib, administrated to IC30 doses for 72h. Tumor cell growth was reduced. Quantification of ATP was used to measure reduction of cellular growth, expressed as % of CTL. Each experiment is representative of three independent experiments. Statistically significant results are reported (*** indicates P < 0.0005, ** indicates P < 0.005 and * indicates P < 0.05). **c** The exposure to fixed dose (IC50) of ipatasertib and taselisib induces the reduction of expression of phospho-mTOR, associated with the increase of autophagy signaling, as showed by increase of LC3 II/LC3 I ratio by immunoblot assay by reduction of p62 and increase of ATG5 after 24h of exposure in MDAMB361 cells (*** indicates P < 0.0005, ** indicates P < 0.005 and * indicates P < 0.05). **d** The addition of CQ 10µM to ipatasertib and taselisib (IC50) induces accumulation of LC3II and p62 protein expression after 24h, due the reduction of autophagic flux, while expression of cleaved parp was not significant increased in taselisib +CQ and in ipatasertib+CQ groups in MDAMB361 cell line. **e** Representative confocal images of MDAMB361 cell lines immuno-stained with anti-LC3IIb antibody reveals accumulation of autophagosomes (green dots) in treatments with ipatasertib, taselisib, CQ or combinations, due to the induction of autophagy or reduction of autophagic flux exerted by CQ. (*** indicates P < 0.0005, ** indicates P < 0.005 and * indicates P < 0.05). **Fig. S2. Ipatasertib and taselisib induce cell cycle alteration as well as apoptosis in Breast cancer cells.** **a** The reduction in proliferation was also associated with perturbation in the cell cycle, after 24h of treatment with ipatasertib and taselisib at IC30 doses, leading to an increase in the G1/G2 phase depending on the cell lines (MCF7, SKBR3, MDAMB361 and MDAMB231) expressed as % of CTL (**b**) or apoptosis measured by enhancement of Annexin V-FITC and Propidium Iodide, after 24 of treatment at IC30 doses, expressed as % of CTL. Similar representative experiments have yielded similar results. Statistically significant results were reported (*** indicates P < 0.0005, ** indicates P < 0.005 and * indicates P < 0.05). **Fig. S3. Ipatasertib and taselisib induce autophagy in MDAMB231 cells.** Representative confocal images of MDAMB231 cells overexpressing GFP-LC3 and immuno-stained with anti-p62 ab, reveal accumulation of autophagosomes (green dots) that colocalise with p62 (Merge) in CQ treatments, due to the reduction in autophagic flux exerted by CQ. Ipatasertib, taselisib and CQ were administered at a 10µM dose for 24h. Similar representative experiments yielded similar results. Statistically significant results were reported (*** indicates P < 0.0005, ** indicates P < 0.005 and * indicates P < 0.05). **Fig. S4. Chloroquine potentiate antitumor effects induced by ipatasertib and taselisib.** **a** The addition of CQ 1-10-20µM to ipatasertib or taselisib inhibits cell proliferation measured by SRB assay after 48h in MCF7 and SKBR3, and 72h in MDAMB361 on treatments indicated, expressed as % of CTL. Each experiment is representative of three independent experiments. (**b**) The addition of CQ 10µM to ipatasertib and taselisib (IC30 for MCF7, SKBR3, MDAMB361 and 10µM for MDAMB231) causes an increase of cytotoxicity measured by LDH release after 24h, expressed as % of CTL. Each experiment is representative of three independent experiments. Statistically significant results were reported (*** indicates P < 0.0005, ** indicates P < 0.005 and * indicates P < 0.05). **Fig. S5. Taselisib induce anti-proliferative effects via autophagy mediated by ATG5.** **a** ATG-5-siRNA transfection in MDAMB231 cell lines induces a relevant reduction in ATG5 protein expression compared with the control non-transfected or scramble transfected cells within 24 h from transfection **b)** In the SRB-based proliferation assay, TAS+ATG5-siRNA and TAS+ATG5-siRNA+paclitaxel groups showed a significant reduction in the proliferation rate after 72h of treatment. Ipatasertib and taselisib were administered at a 1µM dose and paclitaxel at a 0.5nM dose. Statistically significant results were reported (* indicates p < 0.05).

## Data Availability

All data generated or analysed duing the present study are included in this published article.

## Abbreviations

TNBC: Triple negative breast cancer
PI3K: Phospatidylinositol 3-kinase
mTOR: Mammalian target of rapamycin
PI3KCA: Phosphatidylinositol-4, 5-bisphosphate 3-kinase, catalytic subunit, alpha
PTEN: Phosphatase and tensin homolog
mTORC1: mTOR complex 1
CQ: Chloroquine
HCQ: Hidroxychloroquine
ER: Estrogen receptor
HER2: Human epidermal growth factor receptor 2
SDS: Sodium dodecyl phosphate
PAGE: Polyacrylamide gel electrophoresis
ECL: Enhanced chemiluminescence
IP: Intraperitoneally
TV: Tumor volume
TGD: Tumor growth delay
LC3: Light chain 3
PFS: Progression free survival
