# Supplementary figures and images for: Inhibition of autophagy by chloroquine prevents resistance to PI3K/AKT inhibitors and potentiates their antitumor effect in combination with paclitaxel in triple negative breast cancer models
Source: J Transl Med. 2022 Jun 27;20:290. doi: 10.1186/s12967-022-03462-z (PMC9235112; doi:10.1186/s12967-022-03462-z)

Fig. S1

MDAMB361

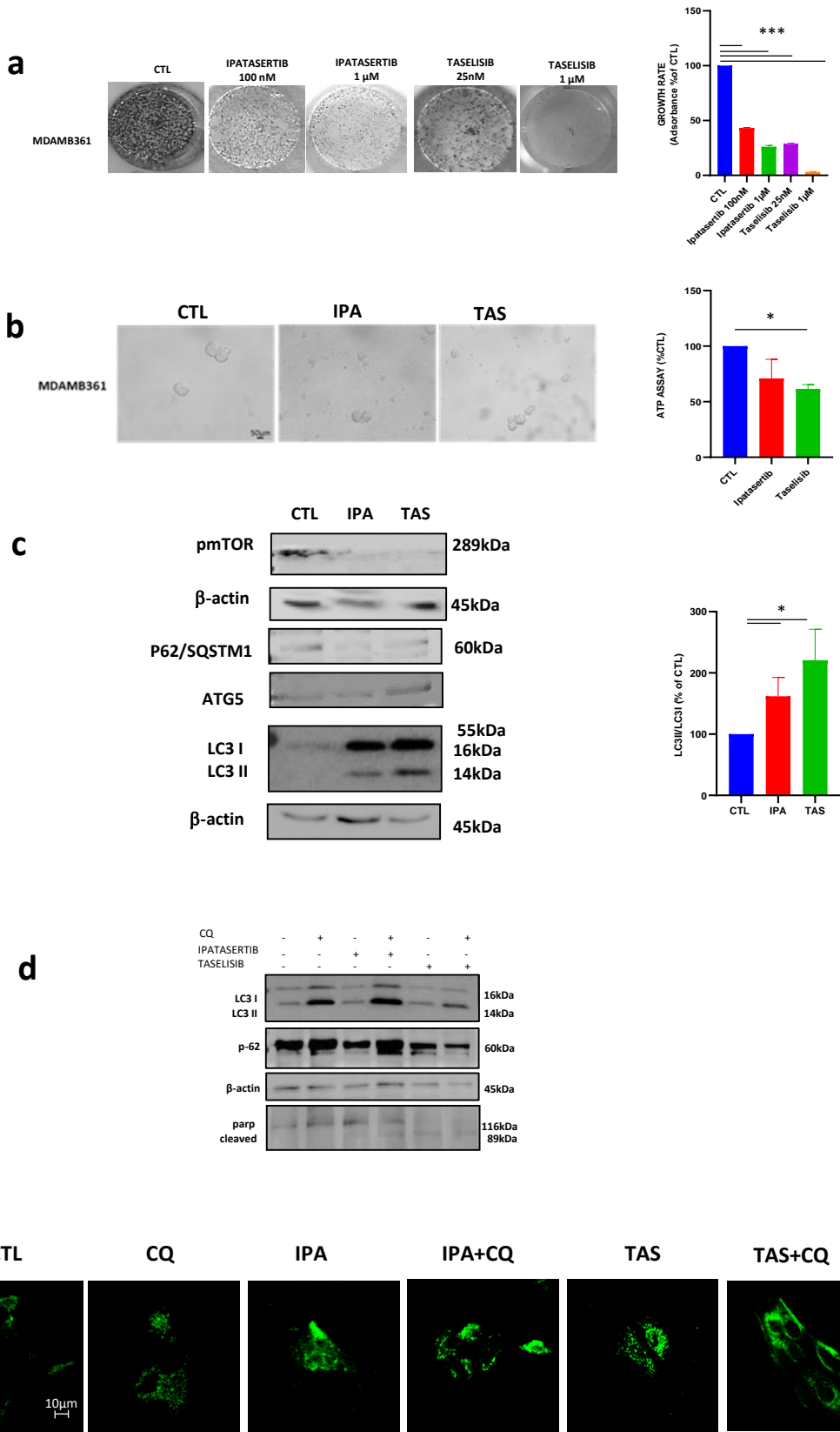

Fig. S2

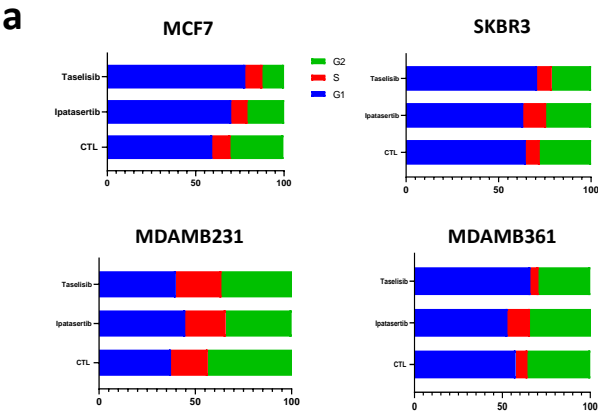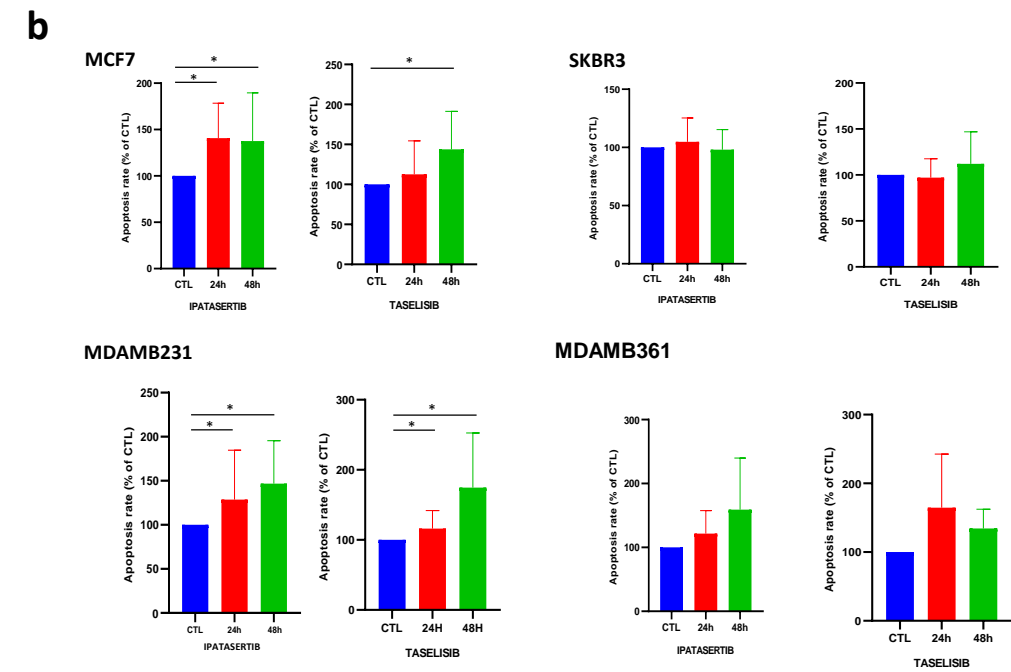

Fig. S3

MDAMB231

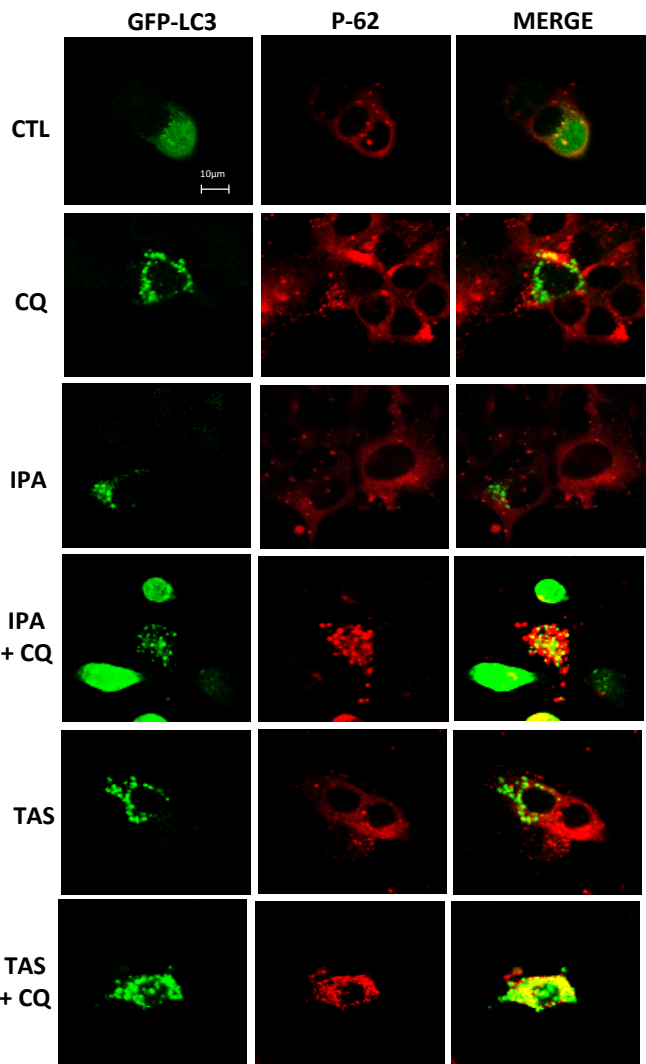

Fig. S4

a

MCF7

SKBR3

MDAMB361

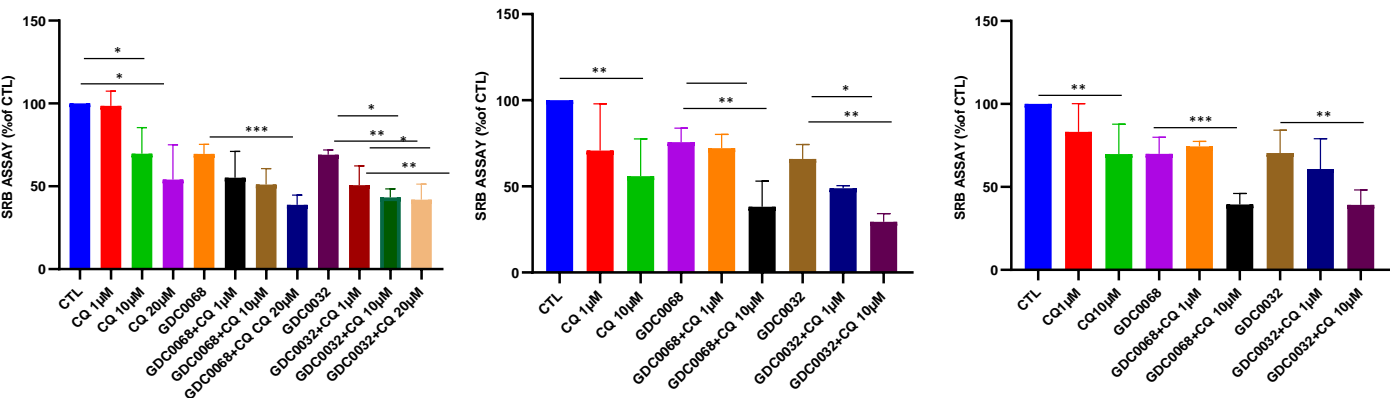

b

MCF7

SKBR3

MDAMB231

MDAMB361

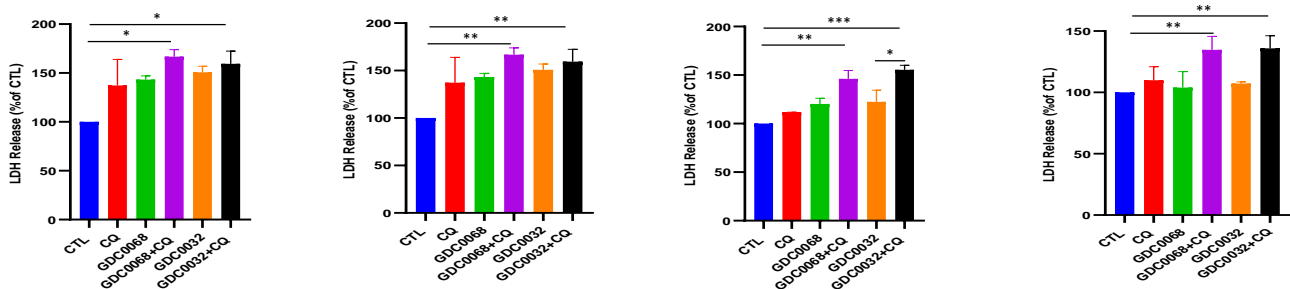

Fig. S5

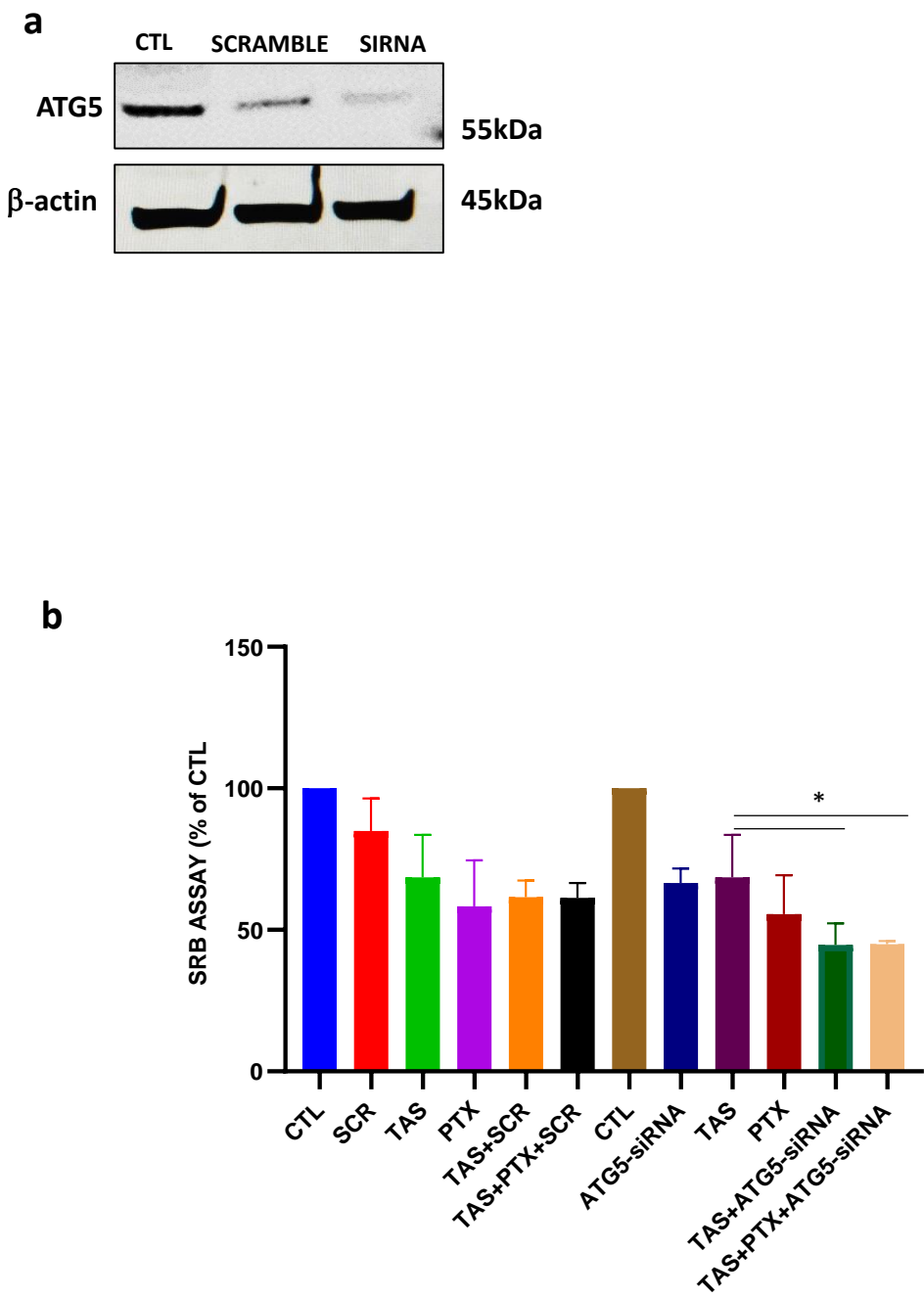

Supplement: Supplementary file 1 — Additional file 1. Fig. S1. Ipatasertib and taselisib determines antitumor effects in MDAMB361 cells. a Ipatasertib and taselisib reduce cell proliferation and ability to prevent clonogenic formation after daily administration for 10 days of IC30 doses for each drug or at fixed drug dose of 1µM, expressed as % of CTL. Each experiment is representative of three independent experiments. b Ipatasertib and taselisib treatment impair 3D tumor spheroid derived from MDAMB361 breast cancer cells. Representative images of first generation 3D tumor spheroid, exposed to ipatasertib and taselisib, administrated to IC30 doses for 72h. Tumor cell growth was reduced. Quantification of ATP was used to measure reduction of cellular growth, expressed as % of CTL. Each experiment is representative of three independent experiments. Statistically significant results are reported (*** indicates P < 0.0005, ** indicates P < 0.005 and * indicates P < 0.05). c The exposure to fixed dose (IC50) of ipatasertib and taselisib induces the reduction of expression of phospho-mTOR, associated with the increase of autophagy signaling, as showed by increase of LC3 II/LC3 I ratio by immunoblot assay by reduction of p62 and increase of ATG5 after 24h of exposure in MDAMB361 cells (*** indicates P < 0.0005, ** indicates P < 0.005 and * indicates P < 0.05). d The addition of CQ 10µM to ipatasertib and taselisib (IC50) induces accumulation of LC3II and p62 protein expression after 24h, due the reduction of autophagic flux, while expression of cleaved parp was not significant increased in taselisib +CQ and in ipatasertib+CQ groups in MDAMB361 cell line. e Representative confocal images of MDAMB361 cell lines immuno-stained with anti-LC3IIb antibody reveals accumulation of autophagosomes (green dots) in treatments with ipatasertib, taselisib, CQ or combinations, due to the induction of autophagy or reduction of autophagic flux exerted by CQ. (*** indicates P < 0.0005, ** indicates P < 0.005 and * indicates [file 12967_2022_3462_MOESM1_ESM.pdf]
